# Plant-Based Virus-like Particle (VLP) Vaccines Against Influenza and Hepatitis Viruses

**DOI:** 10.3390/vaccines14100912

**Published:** 2026-10-20

**Authors:** Mehdi Shahgolzari, Kathleen Hefferon, Afagh Yavari, Goabaone Gaobotse, Kabo Masisi, Mehrin Faija, Abdullah Makhzoum, Srividhya Venkataraman

**Affiliations:** 1 Department of Medical Nanotechnology, School of Medicine, Zanjan University of Medical Sciences, Zanjan P.O. Box 45139-56184, Iran; 2 Department of Cell & Systems Biology, University of Toronto, Toronto, ON M5S 3B2, Canada; 3 Department of Biology, Payame Noor University, Tehran P.O. Box 19395-3697, Iran; 4 Department of Biological Sciences and Biotechnology, School of Life Sciences, Botswana International University of Science and Technology, Private Bag 16, Palapye 1234, Botswana; 5 College of Agricultural and Life Sciences, Cornell University, Roberts Hall, 215 Garden Avenue, Ithaca, NY 14850, USA; 6 Independent Researcher, Burlington, ON L7T 0C8, Canada

**Keywords:** plant-made vaccines, influenza virus, hepatitis virus, VLPs, plant molecular pharming, immune responses, murine models, clinical trials

## Abstract

The rapid evolution of pathogens, particularly viruses, poses a continuous threat to global public health, necessitating the development of highly scalable and cost-effective vaccine platforms. Plant-based virus-like particles (VLPs) have emerged as a transformative approach in vaccinology, leveraging the innate capacity of plant systems to produce highly immunogenic, non-infectious, and structurally authentic viral mimics. VLP-based recombinant antigens contained within the plant tissues (particularly in seeds) show better stability and can be transported and stored at ambient temperatures, without the requirement of a cold chain framework, and edible vaccines offer needle-free vaccine administration. Plant-based expression systems use natural cellular structures such as plant cell walls and seed endosperms to encase antigens, functioning as a natural shield against digestion and thermal degradation. Grain host plants are inimitably adapted for prolonged storage owing to natural seed desiccation wherein recombinant antigens accumulate within vacuoles or intracellular protein bodies segregated from active degradation. By simply cultivating more plants, vaccine production can be scaled up while transient plant expression systems speed up production within a few weeks, which is critical for quick responses to emerging new disease outbreaks. By genetic modification, plants can be used to express several antigens, facilitating the development of multi-component vaccines against many diseases at a single shot. Recently, plant-derived VLP vaccines, principally targeting influenza and hepatitis viruses, have been shown to elicit robust immune responses through the expression of their surface antigens in plants such as lettuce, potato, and tobacco (*Nicotiana benthamiana*). Despite their potential, significant challenges persist, including the achievement of consistent dosing, the attainment of high and reproducible expression levels, and the navigation of complex regulatory frameworks for plant-derived pharmaceuticals which remain unsolved. The current review focuses on recent developments in the production of plant-based influenza and hepatitis vaccines and explores the advantages, challenges, and application of such vaccines in combating these infections.

## 1. Introduction

Plants are increasingly being proven to be a powerful platform for manufacturing biologicals, inclusive of vaccines, therapeutic agents and antibodies. This technology has been under development for over a quarter of a century; both transgenic and transient technologies have been used to produce pharmaceuticals of this type. These include the use of nuclear as well as transplastomic transgenic plants, as well as plant virus expression vectors and virus-like particles to temporally express vaccines. These technologies are relatively inexpensive when compared to conventional pharmaceuticals, are highly scalable and do not need cold chain storage, rendering them amenable for Low-to-Middle-Income countries. While much of the research and development has so far remained in academia, a few companies are beginning to commercialize plant-based biologics. Here, we discuss in detail the use of plant-expressed vaccines to combat infectious diseases based on influenza and hepatitis viruses.

The World Health Organization (WHO) evaluated that influenza viruses kill nearly 250,000–500,000 people per year [1]. There is a shortfall of influenza vaccines worldwide and hence there is dire requirement for “rapid response” vaccines against the pandemic influenza virus strains [2]. The technology of plant molecular farming can address these requirements as it enables rapid development and manufacture of vaccines in addition to scalable production. Recombinant influenza subunit proteins induce poor immune response [3,4,5] due to the lack of structural complexity and danger signal characteristics of whole viruses. Isolated influenza subunit proteins like hemagglutinin are soluble and small and do not generate repetitive particulate arrays (Pathogen-Associated Molecular Patterns: PAMPs) that efficaciously cross-link the B cell receptors and trigger potent antibody reactions. Unlike VLP-based vaccines, purified recombinant proteins do not trigger innate immune receptors, whereas VLPs imitate the structure of the influenza virus while being bereft of the internal nucleic acid. This makes VLPs non-infectious and safe in addition to their capability of stimulating both cellular and humoral cellular immunity [6,7]. VLPs possess assembled repeats of the influenza structural proteins towards optimal presentation and display to antigen-presenting cells [8]. VLPs’ nanostructure serves as a natural booster, thus dispensing with additives such as adjuvants required by recombinant protein antigens. Plant-derived quadrivalent influenza VLP vaccine (QVLP) reached phase III clinical trials, which authenticated the immunogenicity and safety of this vaccine [9]. This demonstrated that the Medicago-generated plant-based influenza vaccine can be expressed in a time span of a few weeks, which is more favorable when compared to cell-based and egg-based expression platforms that need nearly 4–6 months for production [10].

During the year 2009, Kentucky Bioprocessing, Medicago Inc., Fraunhofer USA and the Center of Molecular Biotechnology companies produced 100 million plant-derived influenza vaccines within a span of 1 month [11]. Particularly, they employed transient antigen expression vectors [12] enabling the synthesis and accretion of large amounts of recombinant antigens in plants within a short one-week period. Medicago generated 10 million doses of the influenza H1N1 vaccine within a single-month “rapid fire test” [11]. Medicago Inc. has gainfully passed phase III clinical trials of its plant-made quadrivalent flu VLP vaccine (NCT03739112, NCT03301051, NCT03321968) [13]. This proven high efficiency of plant-derived flu vaccines is a major milestone in the advancement of plant molecular farming.

Hepatitis viruses are a category of viruses principally affecting the liver [14,15]. Hepatitis poses a health challenge on a global scale due to factors including lack of public awareness, asymptomatic infections and circumscribed availability of vaccinations and therapy in developing countries. Recently, plant-derived vaccinations using plant transformation and genetic engineering technologies have been developed to prevent hepatitis A infections. These vaccinations are better than conventional vaccinations in terms of lowered costs, high scalability and increased stability.

Recent developments have been witnessed in the expression of hepatitis and influenza antigens in various plant systems such as rice and potatoes, which afford facile scalability and promise for edible vaccines, thus streamlining the process of vaccine administration and augmenting accessibility. For example, a clinical trial showed that oral, potato-derived hepatitis B vaccines successfully boosted protective antibodies in most previously vaccinated human volunteers [16,17]. Nevertheless, in spite of these favorable developments, the transition of success at the laboratory level to that of clinical trials and the commercial approval and use of these vaccinations have been slow. Challenges like poor yields, the requirement of stable and consistent antigen expression in plants and the accompanying regulatory hurdles still remain major obstacles. Additionally, the efficacy and immunogenicity of these plant-made vaccines require validation in human clinical trials that are still circumscribed. The current review addresses major developments in the development of plant-made vaccines against influenza and hepatitis viruses and serves as a good theoretical source of recent information on plant-made vaccines to combat these emerging viruses.

## 2. Influenza and Hepatitis Viruses

Viral infections are the most considerable threats to public health on a global scale with imposing socio-economic pressures [18]. The most common among them are the infections caused by influenza and hepatitis viruses with their high morbidity and mortality rates. Influenza viruses from the *Orthomyxoviridae* family constitute enveloped viruses, having segmented, single-stranded negative-sense RNA genomes, and are classified into four types: A, B, C and D (Table 1). The influenza A viruses are further classified into subtypes according to their surface glycoproteins, hemagglutinin (HA) and neuraminidase (NA) [19]. Influenza A and B viruses are the primary causative agents of seasonal influenza outbreaks in humans [20]. Influenza viruses also possess pandemic potential due to antigenic drift via gradual mutations and antigenic shift through major genetic reassortment events [21,22]. These mechanisms enable influenza viruses to escape host immune systems and lead to the emergence of novel viral variants [21,22,23]. The genome structures of influenza viruses contain segments (A and B with eight and C and D with seven segments [19]). The influenza A viruses are further categorized into subtypes based on their surface glycoproteins, neuraminidase (NA) and hemagglutinin (HA). HA mediates viral attachment to epithelial cells within the upper respiratory tract, while NA facilitates cleaving progeny viruses and preventing virion aggregation. So far, 11 NA and 18 HA subtypes have been discovered. Influenza B viruses are categorized into two B/Victoria and B/Yamagata distinct lineages.

Hepatitis viruses comprise a diverse group of hepatotropic viruses that principally infect liver cells and cause inflammatory liver diseases [24,25]. The hepatitis A, B, C, D, E, and G viruses belong to distinct viral families, having unique genomic organization, replication strategy and morphology (Table 1) [26]. They are the causative agents of the majority of the global cases of chronic and acute hepatitis. Hepatitis A virus (HAV) and hepatitis E virus (HEV) elicit acute disease only with no chronic sequelae, hepatitis B virus (HBV), hepatitis C virus (HCV) and hepatitis D virus (HDV) cause variable degrees of liver injury and chronicity, which can progress to liver cancers and cirrhosis [26]. Hepatitis A and E viruses are non-enveloped while B, C and D viruses are enveloped. HAV, HCV and HEV have single-stranded positive-sense RNA genomes, HBV has a partially double-stranded DNA genome and HDV consists of a covalently closed circular single-stranded negative-sense RNA genome. HAV and HEV are commonly transmitted through the fecal–oral route, and HBV and HCV are mainly transmitted through blood and bodily fluids. HBV replicates through reverse transcription of an RNA intermediate, the pregenomic RNA (pgRNA) [27]. HDV is known as a “satellite virus” because it can only infect people who are also infected by the HBV [28].

The persistent global burden of influenza and viral hepatitis underscores the need for improved prevention, diagnostic, and therapeutic strategies. Advances in molecular virology, immunology, and biomedical technologies have facilitated the development of innovative antiviral approaches, including nanoparticle-based vaccines, targeted drug delivery systems, and advanced biosensing platforms for early detection. These emerging strategies aim to overcome limitations associated with conventional antiviral therapies, such as drug resistance, limited bioavailability, and suboptimal immune responses. Vaccination plays a critical role in precluding viral infections, especially for diseases such as hepatitis and influenza.

## 3. Virus-like Particle (VLP)-Based Vaccines Against Influenza and Hepatitis Viruses

**Figure 1:**
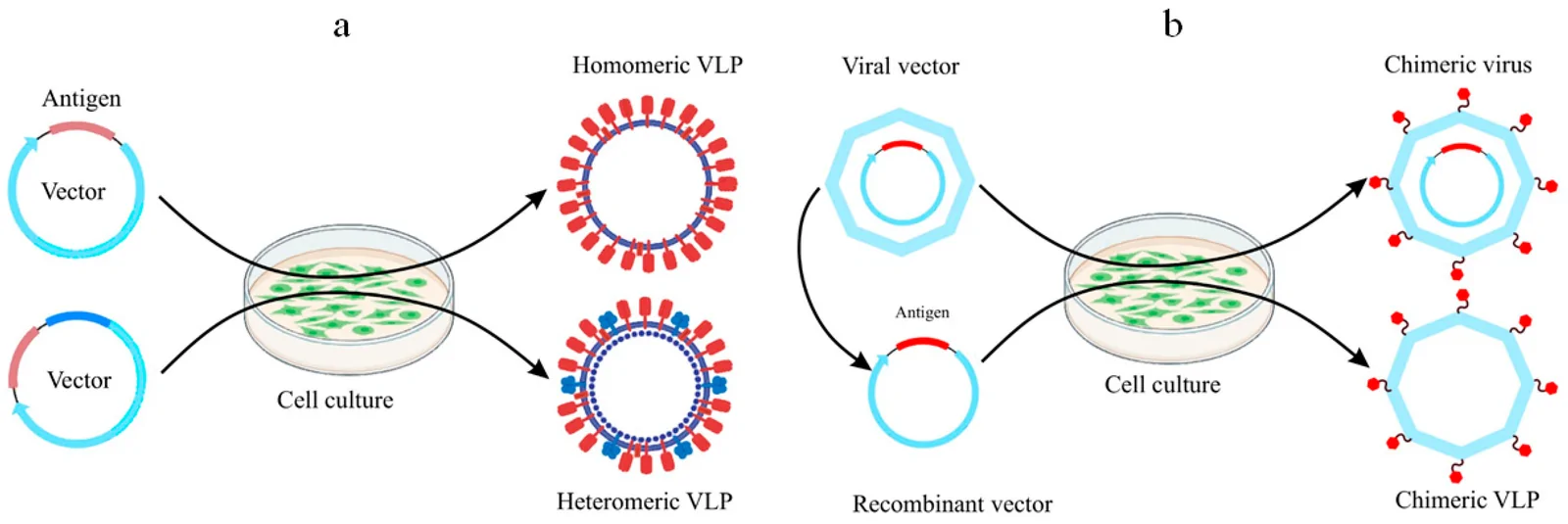
(Own figure) Methods for universal VLP vaccine generation. (**a**) The production of homo/heteromeric virus-like particles (hom/hetVLPs). VLPs generated by cloning of gene sequences of virus antigens (red and dark blue) into the expression vector succeeded by transfection into host cells. (**b**) The production of chimeric virus and chimeric VLPs. Antigen genes (red) are placed into the viral vector to express chimeric viral vectors and chimeric VLPs.

Vaccines are the most cost-effective strategy to combat viral infections. Vaccines can prevent morbidity and occasional mortality and reduce outbreaks associated with viruses. They can significantly reduce the disease burden of influenza and hepatitis viruses on a global scale. However, they are still facing huge issues for some infectious diseases and starting vaccination campaigns [29]. Conventional vaccines are often produced as inactivated viruses and live attenuated viruses with adjuvants or non-adjuvants using egg technology [30,31,32]. The current egg-based system is not flexible in the quantities of doses which can result in vaccine shortages, particularly in pandemic situations [33]. Furthermore, they pose issues such as systemic or local allergic reactions to egg-based vaccine components, human safety concerns, and the supply shortage of eggs for vaccine manufacture [34]. Traditional subunit vaccines with small antigenic parts of infectious viruses could receive a particular interest for immunization. They are composed of immuno-dominant peptides that lack any risk due to the absence of the pathogen and, elicit an immune response [35]. However, poor immunogenicity and the need to produce multiple doses and nontoxic adjuvant formulations can limit their application [36,37,38]. The typical examples of subunit vaccines were those developed against influenza and hepatitis viruses. However, the high mutation rate of the influenza virus, particularly through antigenic shift and antigenic drift, and the protective immune response that is strain-specific, frequently reduce vaccine efficacy [39]. Major challenges remain, inclusive of circumventing immune subdominance associated with conserved regions, providing long-lasting immunity, and ensuring potent correlates of protection [40]. In the case of hepatitis viruses, HAV and HBV have effective vaccines, but there is no licensed vaccine for hepatitis C due to its genetic variability and immune evasion mechanisms [41]. Furthermore, they also pose inherent issues of weak immunogenicity, shortened half-life, and protection that is strain-specific Thus, researchers have greatly focused on resolving these issues. Recently, virus-derived proteinacious nanoparticles, called virus-like particles (VLPs), have been introduced as a versatile strategy [42]. VLPs mimic the conformational and structural characteristics of viruses, without genetic material. They are non-infectious particles with antigenic properties for vaccine development. They can be produced by the heterologous expression systems like insects, bacteria, animals, and plants. VLPs can self-assemble with a single protein (homomeric VLPs, homVLPs) or multiple proteins (heteromeric VLPs, hetVLPs) of a given virus. They are produced by cloning of gene sequences of virus proteins into expression vectors followed by transfection into heterologous expression systems wherein their co-expression allows self-assembly of proteins as VLPs. Thus, cloning of gene sequences of proteins from influenza and hepatitis viruses into expression vectors followed by transfection into expression systems can produce hom/hetVLPs. VLPs can also self-assemble by proteins derived from various viruses into a single structure as chimeric VLPs (cVLPs). The protein encoding genes of a virus are inserted into the virus vectors to express proteins as carriers (Figure 1). Thus, cloning of gene sequences of influenza and hepatitis viruses into the other viral vectors (insect, plant, human, animal) followed by transfection into expression systems can produce cVLPs.

hom/hetVLP-based vaccines have been produced using virus proteins to protect against influenza and hepatitis viruses (Table 2). VLPs are obtained from the envelope protein, the coat protein, or both such as HA, NA or matrix protein 1 (M1), of influenza viruses, and capsid proteins or surface antigens of hepatitis viruses such as large, middle, and small hepatitis B surface antigens (HBsAg) [43]. These recombinant proteins are widely expressed by the various vector systems in cultured cells [44]. Upon expression, structural proteins can undergo oligomerization and self-assemble into VLPs imitating the structure of the native virus [45]. The baculovirus and lentivirus are the best vector systems for producing VLP-based vaccines against viruses such as Chikungunya, Zika, Japanese encephalitis and Yellow fever viruses [46,47]. Lentivirus-based vectors were constructed and used to integrate the complete sequence of core, E1, E2, and P7 genes of the genotype 4 of HCV for expression in Human Embryonic Kidney cells (HEK293T). Upon expression, HCV structural proteins could oligomerize as well as self-assemble into VLPs imitating the structure of native HCV [45].

The advancement of VLPs as platforms for display of foreign antigens (cVLPs) has further widened their potential use as vaccines in protection against diseases [56]. Different virus candidates, including animal, insect, plant and bacterial viruses, have been employed as platforms to develop cVLP vaccines to combat viral infections [45]. The cVLP vaccines can be produced via a genetic fusion technique for co-expression of epitopes on the VLPs into expression systems [57]. Furthermore, the epitopes can be conjugated on the VLP surface via addressable groups (e.g., lysine (Lys) residues) by using chemical techniques [58]. In this regard, the foreign antigens of influenza and hepatitis viruses can use other viruses as scaffolds to display antigens for producing cVLP vaccines [47]. For example, truncated hepatitis E virus coat protein was utilized as a carrier to display four tandem copies of influenza A M2e, and the resulting particles formed cVLPs in plants [59]. The long alpha helix (LAH) domain from the H3N2 influenza strain has been integrated into cVLPs obtained from hepatitis B virus core protein (HBc) by the use of recently designed tandem core technology. Immunologic studies indicate that HBc cVLPs harboring the LAH antigen represent a propitious component of universal influenza vaccine [60]. Furthermore, it has been shown that the HBsAg of HBV can self-assemble into VLPs and be used as a vaccine. The HBsAg proteins can carry foreign epitopes to allow particle formation composed of HBsAg/epitope VLPs [61,62]. Insertion of the hypervariable region 1 (HVR1) sequence derived from the envelope protein 2 (E2) of HCV into the major antigenic site of Small-HBsAg (HBsAgS) resulted in the formation of highly immunogenic VLPs that retained the antigenicity of the inserted HVR1 sequence [61]. Although VLPs based on influenza and hepatitis viruses can be used for the presentation of foreign antigens, VLPs from other organisms can also be used to develop cVLPs, including human papillomavirus [63,64], insect baculoviruses [65]) and plant viruses (PVs, PV-cVLPs) such as Tobacco mosaic virus (TMV) [66] and Physalis mottle virus (PhMV) [67]. The first example of such cVLPs was TMV particles used for vaccine development [66]. Some VLPs can elicit a stronger immune response than others and this fact is very important when choosing the VLP. Plant viruses and their VLPs are a favorable choice due to their highly organized, repetitive spatial structure, their inability to cause infections in human and animals, and their inherent adjuvant properties.

## 4. Plant Expression Systems for Virus-like Particle (VLP) Vaccines

Plants are efficient bioreactors for the production of important pharmaceutical proteins such as vaccines and monoclonal antibodies [68,69]. These systems have been proposed due to their low production cost, high scalability, possibility of suitable post-translational modifications, and low risk of contaminations [70,71]. Plant expression systems can allow the production of chimeric agents in a short period of time (within 1–2 weeks) that can be employed for rapid and scalable applications [72]. The most common plant hosts for transient expression of proteins include tobacco and the related *Nicotiana benthamiana* (*N. benthamiana*) and lettuce (*Lactuca sativa*) owing to their robust growth and ability to be cultivated readily and produce large amounts of biomass rapidly [70].

Plant-based VLPs are appealing production platforms for cost-efficient, scalable expression of vaccines against several human diseases while being bereft of mammalian pathogens. Employing “humanized” plants has enabled circumvention of the challenges of post-translational modifications [73,74]. Plant expression platforms are typified by increased yields of proteins, facile protein purification and rapid production of recombinant proteins [75,76,77,78,79]. Whole plants function as biofactories that can be grown in substantial quantities inside greenhouses without the requirement of fermentation methods based on bioreactors [80]. For plant-based platforms, it is only essential to increase the cultivation area to produce increased amounts of antigens [81]. Also, plants have brief growth cycles in addition to maturing quickly, thereby decreasing production costs in comparison to other systems. Plant viral nanoparticles are also employed as carrier particles for the delivery of drugs as well as imaging [82]. Plant-based expression systems dispense with contamination issues due to endogenous mammalian pathogens and toxins typical of mammalian cell-derived and bacterial systems respectively [83,84,85]. Dissimilar to prokaryotic systems, plants exhibit the same secretory pathway as their human counterparts [86]. Additionally, plant expression platforms afford simplicity of storage of recombinant proteins [87,88].

Plants including rice, carrots, soybeans, corn, tomatoes and potatoes have been explored to investigate immunogenicity, specifically that of oral vaccines triggering mucosal immune responses [89,90,91]. Nevertheless, *N. benthamiana* plants are the most dependable plant expression systems affording several advantages such as high growth rate, cultivability in greenhouses that decrease the risk of contamination spread, availability of appropriate and efficient vectors for augmented gene expression and by virtue of their non-food crop status [92,93,94,95]. Transient expression in plants enables quick, low-cost and high-volume vaccine production [84], while transgenic expression affords stability but yields low expression levels in comparison to transient expression [96]. Transient expression renders screening and generation of selected proteins within a few days that is specifically significant for vaccine development at the time of epidemics. Also, the expression of heterologous proteins is circumscribed to the infiltrated tissue precluding the risk of foreign gene transfer to the plant gametes, thus impeding unintended cross-pollination and dissemination. Further, plant cell culture serves as an alternate bioproduction platform for expression of recombinant pharmaceuticals.

Plant systems have been employed to produce VLPs of human and animal viruses, including Norwalk, Ebolavirus, and hepatitis B core antigen [97,98,99]. These VLPs were produced in tomato, potato, lettuce and tobacco with a structure analogous to natural particles [100], and administered as an edible vaccine [17,100]. Preclinical and clinical studies showed that oral HBcAg (Hepatitis B core Antigen) VLPs plant vaccines with and without adjuvants can increase HBc-specific serum IgG as well as intestinal IgA [100,101]. For example, oral administration of tobacco-produced HBsAgS increased the titer of IgG antibodies in the serum of humans and mice [16,102,103,104,105,106]. Berardi et al. demonstrated that HBcAg VLPs are physically stable under acidic gastric conditions (low pH), but they undergo extensive degradation and agglomeration when exposed to the enzyme pepsin. These particles exhibit greater stability in the intestinal environment and, despite partial protein digestion, they retain their structural integrity and immunogenic epitopes [107]. A plant-based quadrivalent VLP candidate used as an influenza vaccine was able to elicit both strong cellular and antibody responses in the first phase III efficiency study and is presently being finalized by Health Canada [97,108]. One of the most successful and significant examples is Medicago’s plant-derived influenza VLPs for expressing the hemagglutinin (HA) protein in *N. benthamiana*. The plant expression system allowed for high-yield production of clinical-grade VLPs in less than a month, showing broad-spectrum immunogenicity and a favorable safety profile in humans [108].

The milestones of plant expression systems in the development of recombinant plant vaccines were the use of plant viruses and PV-VLPs. Recently, various plant viruses have been utilized to create a platform for expression of antigens on their surface, inclusive of TMV, alfalfa mosaic virus (ALMV), cucumber mosaic virus (CMV), cowpea mosaic virus (CPMV), potato virus X (PVX) and papaya mosaic virus (PapMV) [109]. They are more stable at harsh environments (temperatures, pH) with the antigen expression in large quantities in native plant hosts [97,110]. For example, a vaccine capable of blocking the transmission of malaria and consisting of the *Plasmodium falciparum* Pfs25 surface protein conjugated to ALMV has been produced. This PV-VLP, the Pfs25 VLP-FhCMB, was generated in *N. benthamiana* by transient expression and its immunogenicity and safety in a phase I clinical study was efficiently evaluated [111].

## 5. Plant-Derived VLPs Targeting Influenza Viruses

Plant-derived VLP vaccines against influenza (Table 3) can be generated from Agrobacterium-enabled transient expression of the HA proteins of influenza virus in *N. benthamiana*. Plant-based H5 or H1 VLPs mimic the structure of the virions of influenza to a certain extent while being immunogenic and capable of eliciting both cellular and humoral immune responses [112,113,114]. Each VLP contains only HA occurring as 30–50 homotrimers embedded in an envelope of the lipid bilayer derived from the plant cell [115]. A plant-made VLP vaccine was derived from only the HA protein of H7N9 A/Hangzhou/1/2013 influenza virus [116]. Single immunization with this H7-only adjuvanted vaccine triggered potent humoral reactions and protected murine models against lethal challenge of the virus. A double dose of the unadjuvanted vaccine markedly augmented humoral response and led to 100% protection with major decreases in clinical symptoms, resulting in almost asymptomatic infections. In ferret models, a single round of immunization with this H7 VLP vaccine adjuvanted with alum elicited robust humoral and cell-mediated immune reactions involving CD3+ T cell antigen-specific immune activation. Therefore, this plant-derived H7 vaccine elicited protective immune responses following inoculation with either two unadjuvanted doses or a single adjuvanted dose of this vaccine [116]. Conceivably, the plant-made transient expression system enables generation of VLP structures containing only the HA antigen without the presence of other influenza-derived proteins. A plant-based seasonal quadrivalent VLP vaccine has been studied and shown to trigger cross-reactive T cell and antibody responses in healthy adults [108]. The proven safety of this VLP vaccine in humans propounds that it is a favorable alternative production methodology for generating efficacious and HA-strain corresponding influenza vaccines.

Makarkov et al., 2019, reported plant-based VLP vaccines generated through Agrobacterium-enabled transient expression of the HA protein of influenza in *N. benthamiana* plants [117]. HA-bearing influenza VLPs spontaneously self-assemble and do not need accessory proteins to enable their budding from within the plant cells. These plant-based HA-VLPs measure ~100 nm in size [118]. Every VLP has 30–50 HA spike homotrimers introduced into a plant cell-derived lipid bilayer envelope [114,119]. The HA monomers presented on these VLPs measure ~72 kDa in size, which corresponds to the uncleaved precursor HA0 form [118]. The plant-based H1 proteins possess six N-glycosylation sites, present within the HA1 globular head or in the region of the HA2 stem that harbor hybrid or complex glycans harboring epitopes with core α(1,3)-fucose or β(1,2)-xylose [115]. This plant-derived transient expression methodology enables large-scale, rapid generation of influenza vaccines based on HA at a comparatively low cost, which addresses many of the hurdles (scalability and pace) for production of influenza vaccines in the event of a pandemic and therefore serves as a good alternative to the presently available production schemes for seasonal vaccines [118]. Plant-based VLPs reiterate the vital aspects of native virions of influenza like sialic acid-enabled adherence as well as internalization by the target cells, in addition to VLP envelope fusion with the endosomal membranes and quick elicitation of innate immune reactions [113,114,120]. These plant-derived vaccines have been demonstrated to trigger robust, cross-reactive antibody reactions against both pandemic and seasonal strains of influenza in human trials and animal models [108,121,122]. Further, they elicit cross-reactive and polyfunctional HA-specific CD4+ T cell reactions [108,121,123]. The plant-based VLPs harboring the influenza HA protein are pleomorphic, particulate molecules analogous in size to the native virions of influenza. The molecules of HA present on the H1 VLPs organize into supramolecular complexes, while the soluble H1 protein comparator molecule is monomeric.

The health hazards of influenza rise gradually with age wherein >70% of the fatality related to seasonal influenza outbreaks happens in individuals over age 65 years [124]. Regrettably, the efficiency of the present influenza split-virion vaccines commonly decreases as people get older. Despite vaccines devised for this populace (MF59-adjuvanted, high dose), their efficacy can still be negligible [125]. One parameter that possibly contributes to diminished vaccine efficacy in older individuals is that vaccines utilized in the elderly and adults have all been maximized for antibody generation, particularly antibodies reacting in the hemagglutination inhibition assay. Recently, it has been found that in older adults, protection for influenza is afforded principally by cellular responses rather than antibody reactions [126,127,128]. Despite the sub-optimal protection provided by the existing vaccines, elderly individuals can derive advantage from the seasonal influenza vaccines wherein poor antibody response occurs [129,130,131]. Other elements contributing to the poor vaccine efficacy in those who are elderly are the accretion of comorbidities and chronic inflammatory conditions progressively with age in addition to immunosenescence which manifests as gradual debilitation of several arms of the immune system [132,133]. The comorbidities in the elderly include ailments such as diabetes, degenerative diseases, arthritis and cancer. There is an obvious necessity to generate influenza vaccines that afford greater protection in the increasingly elderly populace on a global scale.

The trimeric H7 transiently expressed in *N. benthamiana* was successfully conjugated onto nanodiamond particle surfaces [134]. Following 2–3 immunizations in murine models, the mixture of nanodiamond and trimeric H7 protein triggered statistically significant robust IgG immune response specific to H7 proven by the increased quantities of H7N9-specific IgG.

Avian influenza virus (AIV) causes highly infectious viral disease in which some of the subtypes (such as H9 and H5) elicit elevated mortality in fowls in addition to functioning as zoonotic agents with potential to be disseminated to humans [3]. AIV continues to incur major losses in spite of the availableness of commercial vaccines. The principal antigen of influenza is haemagglutinin wherein neutralizing antibodies are triggered against this protein upon infection. VLPs imitate the shape of the influenza virus while being devoid of the internal genetic material, and therefore are non-infectious but trigger a potent immune response. The H5 VLPs when administered as a single dose in chickens elicited antibody responses capable of neutralizing virus infectivity. This study showed that plant-made vaccines are a propitious alternative to conventional vaccines, particularly in developing nations. For instance, the highly pathogenic avian influenza (HPAI) viruses belonging to the H5 subtype have been disseminating in Egypt for nearly 20 years. Over the past ten years, H5N1 viruses of the clade 2.2.1 have been followed by the antigenically different H5N8 viruses of clade 2.3.4.4 [3]. Additionally, the H9N2 avian influenza viruses co-circulate along with H5N8 viruses among Egyptian poultry. It has been by and large recognized that efficacious vaccination against inactivated avian influenza viruses necessitates a close match of antigens between the vaccine and the respective viruses that circulate in the field. Hence, strategies to generate cost-efficient vaccines capable of rapid adaptability to local strains of the virus are needed for developing nations like Egypt. The Egyptian H9 and H5 virus HA proteins were generated by transient transfection of the *N. benthamiana* plants [3]. Mice were administered with four doses of H9 or H5 VLPs along with adjuvant which led to cellular and antibody responses. Chickens administered with a single dose of the H5 VLPs elicited HA-specific antibodies. This proved that plant-derived VLP vaccines have the ability to function as efficacious vaccine candidates within a brief time period at comparatively low cost.

**Table 3:** Plant-derived VLP vaccines against influenza viruses.

Target Virus	Viral Antigen (Target)	Plant Expression System	Outcomes	Ref
IAV	M2e	*N. benthamiana*	Induced high levels of M2e-specific IgG antibodies in serum and mucosal secretions	[135,136]
Avian influenza A virus (AIV)	Haemagglutinin (HA) proteins	*N. benthamiana*	A high number of interferon-γ secreting splenocytes, isolated from mice. The highest percentage of neutralization in chicken	[3]
Influenza A H7N9	Hemagglutinin	*N. benthamiana*	Induce strong humoral and CMI responses with antigen-specific activation of CD3+ T cells	[116]
H1N1 influenza virus	Hemagglutinin	*N. benthamiana*	elicited robust hemagglutination inhibition antibody responses in mice at doses lower than 1 μg	[49]

## 6. Plant-Derived VLPs Targeting Hepatitis Viruses

Plant-derived VLP vaccines against hepatitis (Table 4) can be generated from Agrobacterium-enabled transient expression of the proteins of hepatitis viruses in plants, especially *N. benthamiana*. For example, HCV-VLPs have been successfully produced in *N. benthamiana*. The HCV core protein has been expressed in tobacco plants utilizing a vector based on TMV [137]. Tobacco plants that expressed the epitope for HCV E2 hypervariable region 1 elicited distinct neutralizing antibodies upon immunization of mice [138]. The capsid protein of papaya mosaic virus was used as a carrier by fusion to a C-terminal epitope of the HCV E2 protein that triggered enduring humoral response (over 120 days) [139]. Tobacco plants were used as expression platforms of the HCV core protein through a PVX-based viral replicative vector and a non-replicative, binary vector, namely pBI121 [140]. The PVX-based vector afforded high expression levels that were augmented by co-expressing the P19 gene silencing suppressor (up to TSP level of 0.022%). Mohammadzadeh et al. (2015) used a PVX-derived viral replicative vector to express a HCV polytope in fusion with the surface antigen of Hep B [141]. Transiently expressed E1-E2 heterodimers of HCV in lettuce induced mucosal and systemic immune reactions in BALB/c female mice upon administration of plant extracts through intramuscular immunization succeeded by two oral booster doses as proved by the detection of secretory anti-HCV IgA in the feces of immunized mice [142].

López-Castillo et al., 2026 [73], reported a pioneering study involving the fruitful expression of complete VLPs derived from HCV in *N. benthamiana.* The HCV structural proteins were expressed by means of both a binary vector system and a deconstructed viral vector wherein co-expression of the HCV E1, core, and E2 proteins in plants was shown to be essential and adequate for self-assembly into VLPs closely resembling the morphology of authentic viral particles. These VLP structures were found to be highly immunogenic which represents a significant advancement owing to their ability to mimic the conformations of the viral surface proteins without harboring infectious genetic material [143].

HBsAg particles were the first VLP-based vaccines and are still commercially manufactured due to being compatible with a wide range of expression systems. Another commercially used VLP is the HBV core particle (HBc), which has been used to produce vaccines against HBV and other infectious diseases. HBc is capable of eliciting strong immune responses, and HBc-VLP can present various foreign antigens, making HBc-VLP a promising platform for vaccine manufacturing [75]. VLP-based vaccines are similar to traditional vaccines in eliciting humoral and immune responses and they also preserve the native structure of the virus. The generation of HBc-VLP-derived vaccines using plants is a favorable option in developing nations where execution of more advanced technologies is often difficult. VLPs displaying H5N1 influenza antigens have been expressed in plants and have been proven to be safe in phase II clinical trials [123]. VLP vaccines expressing the HBcAg as well as PreS1 and PreS2 proteins were shown to trigger potent antibody responses [56]. High-level expression of immunogenic HBcAg has been demonstrated using plants. Many recombinant VLPs inclusive of HBcAg VLPs that display the influenza A epitope and HBcAg VLPs presenting malaria epitopes have been assessed. Further, the expression and formation of VLPs containing HBcAg-HPV16 L2 epitope was reported in tobacco plants which upon administration in mice triggered an antibody response that was antigen-specific [35].

Mardanova et al., 2024 [59], reported plant-based transient expression of VLPs by using a PVX-derived self-replicating vector. In the HEV capsid protein at the Tyr485 position, GFP (green fluorescent protein) and four M2e peptides from influenza A virus were inserted, and the results indicated that both HEV/GFP and HEV/4M2e were successfully expressed and formed VLPs [59]. The GFP and M2e showed evidence of being present on the outer surface of the VLPs, as they were recognizable by their respective antibodies. Earlier studies demonstrated high-level expression of the truncated HEV capsid protein in plants wherein they successfully assembled into VLPs [144,145]. Additionally, this version of the HEV capsid can be employed as a carrier for antigens in plant-based expression systems [146]. Importantly, HEV is better than another renowned antigen carrier for display of foreign peptides, the HBc antigen in terms of foreign peptide presentation, and expression levels [147]. In *N. benthamiana* plants, expression of the HBc and HBc-M2e fusion proteins was reported to be up to 5–10% of plant total protein [148], which is several magnitudes lower than that of the HEV [145]. Mardanova et al., 2020 [145], demonstrated the display of a single copy of the M2e peptide of influenza virus on VLPs derived from HEV coat protein (110–610 amino acids) wherein the recombinant protein was efficaciously biosynthesized in plants and successfully assembled into VLPs. Nevertheless, when mice were immunized with these particles, only low-level elicitation of M2e-specific antibodies was reported. On the other hand, upon linkage of many copies of the M2e protein to the carrier, increase in M2e-specific immune reaction and augmented protective properties of the candidate vaccine were reported [149,150,151,152]. Also, VLP-based expression of M2e peptides in multiple copies expanded the strain specificity of this vaccine. When HEV alone was expressed in plants without any foreign peptides, its expression level was as high as 10–20% of the TSP [145], whereas the level of the HEV/M2e protein containing a single M2e copy was about 10% of the Total Soluble Protein (TSP) [146]. Following purification, the HEV/4M2e protein was expressed at a final yield of 60–80 µg in 1 g of leaf biomass which is high enough for protein expression using a plant-based expression system [59,153].

**Table 4:** Plant-derived VLP vaccines against hepatitis viruses.

Target Virus	Viral Antigen (Target)	Plant Expression System	Outcomes	Ref
Hepatitis C virus (HCV)	Proteins Core, E1, and E2	*Nicotiana benthamiana*	Humoral and cellular specific responses	[73]
Hepatitis E virus (HEV)	The HEV ORF2 capsid protein	*Nicotiana benthamiana*	Induced high levels of HEV-specific serum antibodies	[146]
Hepatitis B virus (HBV)	HBsAg	Lettuce	Developed specific serum-IgG response	[17]

## 7. Plant Virus (PV)-Based Chimeric VLPs Vaccines Against Influenza and Hepatitis Viruses

**Figure 2:**
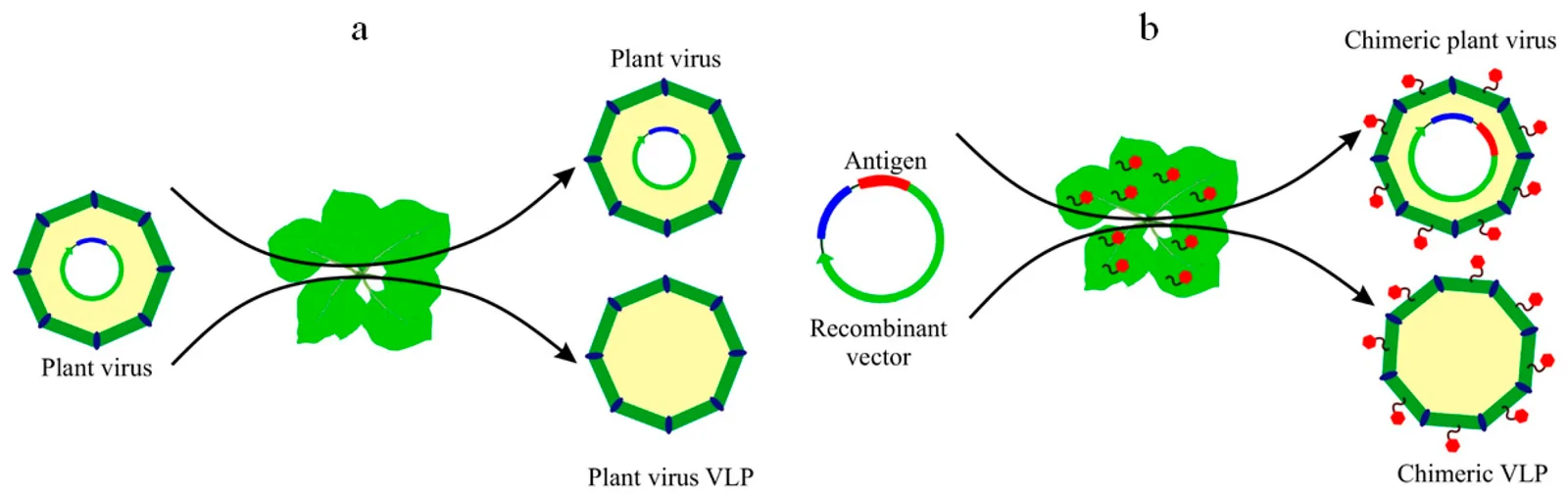
(Own figure) PV-VLP vaccine development. (**a**) The production of native plant virions and VLPs in plants. (**b**) The production of chimeric plant virus and chimeric pVLPs. Plant viral vectors express antigenic protein (red). Antigen genes are placed into the viral genome to express chimeric plant virus or recombinant vector expressed chimeric PV-VLPs.

Plant virus (PV)-based VLPs (PV-VLPs) are self-assembled protein particles from the plant virus coat proteins (CPs) [42,154]. These PV-VLPs lack the functional genome, and they are not infectious and do not replicate in its host and animal cells [155]. CPs of various plant viruses have been considerably investigated for the generation of PV-VLPs [156]. The PV-VLPs obtained from them have garnered attention as biomimetic platforms for encapsulating or displaying therapeutic and imaging agents [156]. The PV-VLPs possess slightly distinct structural and functional characteristics, even among members belonging to the same genus [157,158]. The preference of PV-VLPs is largely dependent on the availability and effort needed to acquire sufficient amounts of high-quality, pure VLPs with the desired properties. The PV-VLPs can self-assemble in the form of spherical, icosahedral, or rod-like structures based on type of virus [98]. The PV-VLPs can be effectively expressed in plants and various systems, inclusive of yeast, mammalian, and insect cell cultures [158]. PV-VLPs can be produced in some plants when they are infected with viruses, especially CPMV (Figure 2a) [159]. Furthermore, the PV-VLPs can be employed as carriers of foreign sequences to display immunological epitopes (Figure 2b) [160]. The epitope sequences inserted or replaced into the CP gene of plant viruses are cloned and expressed in a heterologous expression system [109]. Another approach to creating multivalent vaccines is chemical conjugation of epitopes on the PV-VLPs [161]. Several plant virus vectors, including the geminivirus bean yellow dwarf virus (BeYDV), tobamovirus (TMV)-based deconstructed viral system (*magnICON*) and Cowpea mosaic virus hypertranslatable (CPMV-HT) systems, have been introduced for vaccine development [97,162]. For example, the CPMV-HT platform, which is based on the quick transient expression of heterologous proteins in plants via Agrobacterium-mediated infiltration, has been utilized to produce 10 million doses of VLP H1N1 (swine flu) vaccine within just 30 days in comparison to the 9–12 months needed for more routine approaches in current use [119,163].

Several recombinant pVLPs of influenza (Table 5) and hepatitis viruses (Table 6) with displaying antigens are investigated in medicine as vaccines. For example, the rod-shaped TMV has been engineered to display conserved influenza and hepatitis epitopes, such as the extracellular domain of the matrix protein 2 (M2e), 5B19 epitope [164]. These chimeric PV-VLPs elicited strong, cross-reactive antibody responses in animal models, offering a path toward a universal influenza vaccine. Chimeric PV-VLPs utilizing CPMV [165], ALMV [166], Pod pepper vein yellows virus (PoPeVYV) [167], CMV [168], and PVX [141] have been engineered to present antigenic loops of influenza and hepatitis viruses.

## 8. Immune Activation of VLP Vaccines

VLPs, due to their multimeric structures and conformations resembling wild type viruses, can serve as pathogen-associated molecular patterns (PAMPs) [175,176]. Therefore, VLPs can be recognized by pathogen recognition receptors (PRRs) on the surface of cells or endosomes of the antigen-presenting cells (APCs) [176]. VLP internalization triggers APC maturation and presents vaccine antigens into major histocompatibility complex (MHC) class I for priming CD8+ cell responses or major histocompatibility complex (MHC) class II to prime CD4+ T cell responses [176]. Furthermore, B cells can act as APCs, taking up VLPs, followed by processing and presentation to T cells [177]. The multimeric epitopes on VLP surfaces can induce B cell receptor (BCR) cross-linking, and induce antibody production [176]. Here, B cells operate independently of CD4+ T cells (T cell-independent pathways) [177]. Meanwhile, B cells can be active via follicular helper T (Tfh) cells (Tfh1-, Tfh2 or Tfh17) for producing high-affinity antibodies after vaccination or infection (T cell-dependent pathway) [178]. Tfh cells play an important role in orchestrating B cell differentiation, maturation, and immunoglobulin class switching [179]. Here, different CD4+ helper T cell populations help B cells to express antibodies [156]. Thus, depending on the nature of the trigger, the immune response can generate transient low-affinity IgM antibody responses or high-affinity responses by class switching to IgG. The consequences of these immune reactions can vary widely, from minimal effects to severe and life-threatening outcomes.

VLPs provide an ideal scaffold for multivalent subunit vaccines because of the multivalent presentation of antigens. Investigations utilizing these platforms have demonstrated that increasing antigen valency significantly enhances immune cell activation, which in turn correlates with more robust downstream immune responses [180]. For example, the P22-HAhead is a VLP vaccine consisting of the HA head domain from A/Puerto Rico/8/1934 (H1N1) incorporating the SpyCatcher protein covalently bonded to the P22 bacteriophage coat protein engineered to incorporate a SpyTag [181]. Mice immunized with P22-HA demonstrated superior anti-HA IgG production and HA neutralization titers. The admixture of P22 and HA head, anti-HA IgG and HA neutralization of antisera from this group was not as effective as in the full VLP group. In another study, subcutaneous inoculation of HA from A/Puerto Rico/8/1934 (H1N1) attached to the bacteriophage MS2 via the streptavidin–biotin elicited significantly higher neutralization titers than control animals [182]. Thus, these results showed that stable linkage can be essential to prevent antigen dissociation *in vivo* that can be achieved through genetic fusion or non-chemical (enzymatic e.g., SpyTag/SpyCatcher) methods. On the other hand, while covalent linkage is essential, conventional *in vitro* chemical conjugation can be non-selective and can unintentionally disrupt the protein’s native structure and consequently impact the efficacy of the immune response.

VLPs are appealing as favorable vaccine candidates as they are rapidly transported to lymphatic tissues, in addition to delivering antigen infusion to APCs and subsequent APC activation that results in triggering both cellular and humoral immune responses [108,183,184,185]. Landry et al., 2014, reported that several of the above advantages are featured in plant-derived VLPs that bear HA proteins of both seasonal and pandemic influenza viruses [123]. These VLPs were studied at the preclinical level in young animals as well as in clinical trials involving healthy young adults, wherein they elicited potent, cross-reactive antibody reactions in addition to inducing prolonged, multifunctional CD4+ T cell reactions [108,123]. Hodgins et al., 2019, compared the cellular and humoral responses to the H1N1 A/California/07/2009 H1-VLP vaccine with those of the split influenza vaccine in aged mice having natural comorbidities [124]. The elderly are at increased risk due to influenza, partly as their immunity diminishes with age in addition to the accretion of comorbidities. A novel plant-based VLP vaccine harboring influenza HA antigen can elicit balanced cellular and antibody immune responses in old mice (between 16 and 18 months of age), whereas split influenza virion vaccines trigger mostly antibodies. As mice also accrue comorbidities and undergo loss of immune competence with increasing age, similar to humans, the efficacy of plant-based VLPs was tested in mice nearing the final stages of their lifespan. This revealed that even in mice that are very old and that have comorbidities, the plant-derived H1-VLP vaccine triggered more potent and balanced immune reactions compared to the inactivated influenza vaccine. Further, mice with fewer comorbidities were observed to have better cellular and humoral responses. Taken together, these novel plant-based H1-VLP vaccines are propitious in addressing the drawbacks of current influenza vaccines in aged adults. Inactivated vaccines of influenza virus have endeavored to furnish dependable protection in aged individuals [186]. Bypassing immune senescence involving senescent immune response typified by weak humoral immune reactions to vaccines and unbridled inflammation at the time of infection call for the development of novel immunization regimens. Plant-derived VLPs harboring recombinant HA proteins of influenza have been proven to afford protection in aged animals in studies involving preclinical challenge, in spite of triggering comparatively poor or absent humoral immune responses. Alvarez et al., 2022, report that 3 µg of a plant-based VLP harboring the hemagglutinin of H1N1/California 07/2009 (H1-VLP) when intramuscularly administered as two doses that are 21 days apart produced H1-specific Th2 and Th1 cells accompanied by preclusion of protracted pulmonary inflammation and fatality in both aged and adult mice [186]. In this process, Alvarez et al., 2022, recognized a novel IL-1R1+ tissue-adapted population of regulatory T cells within the lungs of adult and older mice vaccinated with H1-VLP, propounding elicitation of an inimitable regulatory T cell population related to vaccine-associated protection [186]. This investigation offers preclinical evidence wherein the plant-derived VLP vaccine could function in part, inducing both antibody and cellular immune responses, and owing to their multimeric nature, co-administration of adjuvants is not needed in a majority of cases. Nevertheless, the use of adjuvants enhances their immunogenicity.

**Figure 3:**
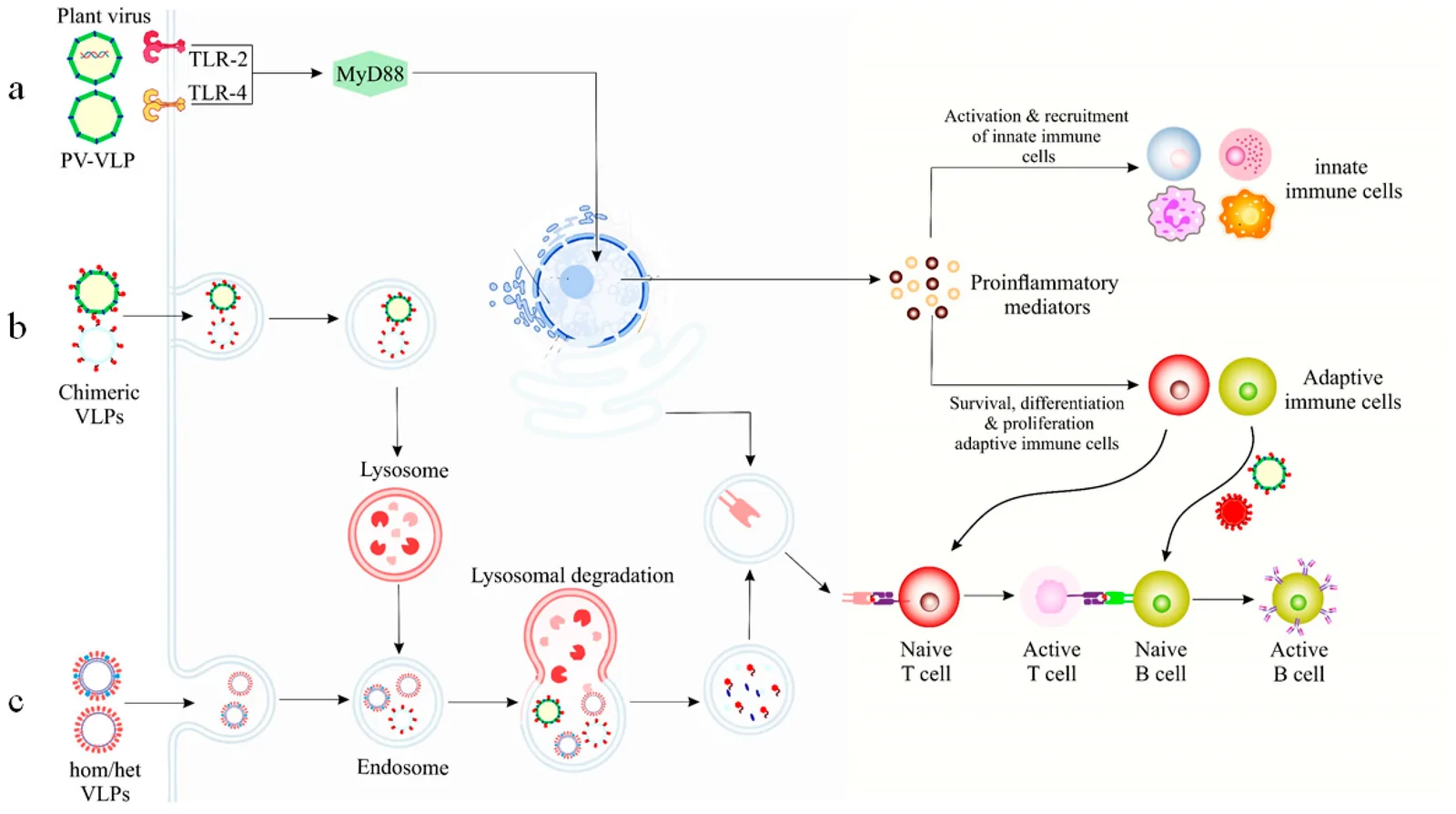
(Own figure) (**a**) Plant viruses (PVs) and their VLPs (PV-VLPs) can activate the innate immune system via TLRs 2 and 4. The pVLPs activate and recruit innate immune cells via proinflammatory mediators. (**b**) Chimeric VLPs release antigens and generate an adaptive immune response. (**c**) The phagocytosed virus antigen-based VLP (homo/heteromeric VLPs) can be processed and presented by MHC class for detection by T cells.

It has been demonstrated that some PV-VLPs (e.g., CPMV) or plant virions (CPMV, ALMV) can induce strong immune responses [187]. The highly ordered, repetitive surface structure of PV-VLPs can facilitate B cell receptor crosslinking, resulting in potent B cell activation and production of antibodies [154]. Moreover, PV-VLPs are easily taken up by the APCs, which process as well as present VLP-derived foreign antigens to T cells, further augmenting the immune response [154,188] (Figure 3). Thus, antigens displayed on the PV-VLP surface interact with APCs that can trigger innate and adaptive immune responses [97]. For example, TMV and CPMV chimeric VLPs, while displaying the foreign antigens, can elicit a robust immune responses in animal models, underscoring their potential as vaccine candidates [189,190].

## 9. Advantages of Plant-Based VLP Vaccines

The importance of plants to humans goes beyond them just being food. Plants provide us with medicinal herbs and different raw materials such as wood. They can also be used to manufacture pharmaceuticals such as vaccines. The advantages of utilizing plants as an expression system for vaccine production is that they are safe and cheap to use and can be scaled up with relative ease. Additionally, plant cells have the ability to perform several post-translational modifications found in animals and people, and this is especially pivotal for immunogenicity [191]. There are several plant-expressed viral capsid proteins that can self-assemble into VLPs. These VLPs do not have the viral genome but possess a size and structural resemblance to natural viruses and this makes them ideal for stimulating strong immune responses in hosts [98].

Traditionally, inactivated pathogens have been shown to produce effective vaccines with very high potency. However, the use of these inactivated pathogens comes with associated safety risks. The primary concern is that if the pathogens are not completely inactivated, they become a danger to humans. In addition, currently, there are a lot of pathogens which do not have safely attenuated strains. Plant VLPs have been shown to have multiple advantages over native viruses, which has encouraged their research and clinical trials. Because plants provide post-transcriptional modifications and are highly scalable and cheap, they provide a very fast and cost-effective alternative to developing virus-like particle (VLP) vaccines.

Some VLP vaccines such as the HBV vaccine have been previously approved by regulatory medical bodies and commercially licensed. Examples of these are Energix^®^ which was produced by the company GlaxoSmithKline (GSK), Rixensart, Belgium and the Merck and Co., Inc, West Point, PA, USA produced vaccine called Recombivax^®^. The efficacy of these hepatitis B vaccines has been reported. They have also been shown to elicit durable antibody responses in human beings [192].

Plant-based virus-like particles (VLPs) afford several advantages because they mimic natural viruses although they do not have the viral genome. Additionally, because VLPs do not have the viral genome, this eliminates the possibility of viral nucleic acid being inserted into the genome in humans. VLPs also eliminate the risk of cancer and prevent unwanted host responses that are commonly associated with vaccines produced by viral vectors [193].

VLPs have been demonstrated to be more stable than subunit vaccines. They can be produced without the need for viral expression using recombinant technology [194]. One of the benefits of VLPs is safety. VLPs are considered safe because they do not have viral genomes and hence cannot replicate. This consequently means that there can never be virulence reversion allowing VLPs to offer a great deal of protection [36]. Additionally, it is very safe to work with VLPs because, since they do not have any genetic material, people handling them are never at risk of any infection. Because the structure of plant VLPs mimics that of native infectious viruses, VLPs can stimulate strong immune responses (both humoral and cellular) without adjuvants [192].

Another asset of plant-based VLPs is their immunogenicity. The immunogenicity of VLPs is a result of their repetitive surface units that induce B cells and the fact that they are particulate [195]. Because they are particulate, VLPs can stimulate T cell-mediated immune reactions by interaction with APCs [194]. VLPs can be substitutes for both live and inactivated vaccines. Generally, VLPs make vaccines that are more effective than recombinant antigens.

Unlike other expression systems, the usage of plants in the expression of VLP vaccines does not need costly equipment such as fermentation facilities which are requisite for biomass production. Additionally, using plants for VLP vaccine production eliminates the need for facilities that are used for scale-up production. In this fashion, the production of plant biomass is less financially intensive [196].

Many clinical trials have proven the immunogenicity of plant-made VLPs for diseases such as hepatitis B virus and influenza. It has been reported that when people are immunized with the HBV VLP vaccine, there is an induction of an antibody response that lasts for a very long time such that even after 10 years of vaccination, that response will still be present [197].

## 10. Challenges and Technical Limitations

There are challenges that affect the use of plant VLPs in vaccine development. These challenges are responsible for the low commercialization rates of VLP-based vaccines. Technical limitations have also impacted progress in the development of VLP vaccines. In order for VLP vaccines to be manufactured successfully at a commercial scale, there ought to be an appropriate culture platform that can permit large-scale manufacturing.

Even though plants such as tobacco have been used in the generation of plant-based vaccines, they possess high levels of toxic alkaloids and phenolics. This makes downstream processing difficult due to technical difficulties [196]. This is partly the reason why lettuce is often preferred because it possesses lower levels of secondary metabolites. One of the challenges in the expression of recombinant proteins in plants is yield. Most often, expression levels of recombinant proteins in plants are variable or low [153]. Recombinant protein purification from plants is also a challenge.

Plant-specific glycans provide an advantage during the production of engineered VLPs with high immunogenicity [198]. Even though there is high conservation of protein synthesis and folding pathways between plants and animals, the glycosylation of proteins within plants has some differences to that in animals. Improper glycoforms may have an adverse effect on VLP vaccine efficacy [198]. Structural variabilities between animal and plant glycosylation pathways pose a major stumbling block in plant molecular farming. If a given plant-expressed therapeutic protein carries improper glycoforms specific to plants, its clinical efficiency can be drastically compromised by alterations in immune-enabled clearance and pharmacokinetics. Plants expressing human therapeutic antigens without modification result in rapid clearance of the antigen in the serum. For human therapeutics such as monoclonal antibodies, the precise architecture of the sugar moieties attached to the Fc region determines how efficiently it interacts with the human immune cells. Plant glycoforms change this structural interface, notably attenuating the therapeutic ability of the antibody to recruit the human immune system to eliminate target cells. Also, neutralizing anti-drug antibodies may be generated by the human immune system resulting in the removal of the therapeutic molecule from circulation prior to achieving its therapeutic objective. Further, pre-existent IgE antibodies against sugars in the plant can elicit adverse allergic reactions or hypersensitivity. Proper folding and glycosylation are critical for the efficiency of VLP-based vaccines as they are important for pathogenicity, immune recognition and stability [199]. The storage of VLP-based vaccines is itself a technical challenge.

Regulatory issues also affect VLP-derived vaccine production. This is because the manufacturing of VLP vaccines involves environmental and safety concerns. Because this involves the production of recombinant proteins, there are concerns about recombinant material entering the ecosystem or the food chain. In countries such as the USA, the manufacturing of pharmaceutical proteins is highly regulated. For example, the Food and Drug Administration (FDA) is heavily focused on the production process itself for vaccines and drug manufacturing, while the USDA (US Department of Agriculture) and EPA (Envionmental Protection Agency) is more focused on the containment of recombinant material [81]. There are regular risk assessments that are done to ensure that humans, and the environment, are not adversely impacted by plant-made proteins and pharmaceuticals.

## 11. Conclusions and Future Perspectives

The 21st century has witnessed extraordinary advancements in the development of plant-made vaccines against several viral diseases. Although an overwhelming preponderance of these developments are still at the laboratory stage while some are at the level of preclinical studies in animal models, the world is also seeing major achievements of companies such as Kentucky Bioprocessing Inc. and Medicago Inc., whose candidate vaccines against SARS-CoV and influenza have advanced to the stage of immunizing humans. Many factors contribute to the observed developments in plant-made vaccines. There is a transformation from the stable expression of viral antigens in transgenic plants to transiently expressed plant-based platforms. Transient expression is favorable as it is more rapid and yields viral proteins within one week following infiltration of plant leaves with an Agrobacterium suspension carrying the target gene of the viral antigen, affording rapid response to the yearly emergence of antigens belonging to novel strains of influenza or unprecedented pandemics. Yet, another significant element for the present advancements in plant-made vaccines is the latest achievement in plant-based systems that not just express the target viral antigens but also enable VLP assembly. Plant-based VLPs are more favorable compared to simple recombinant protein expression in plants as VLPs present antigenic epitopes in their respective native conformations, which lead to increased immunogenicity and superior efficacy as candidate vaccines. In addition to these factors, the viability of the plant-based SARS-CoV-2 vaccine manufactured by Medicago will expedite the generation of other plant-made vaccines in the near future. Time-to-commercialization, the time required for research and vaccine development, scalability, public awareness and regulatory approval influence the choice of plant-based expression systems, wherein time-to-market elements are the major determinants of the recruitment process [200].

In the future, an mRNA-based approach will be applied to plant virus VLP research. In this instance, mRNA vaccines, instead of being enveloped in lipid nanoparticles, are instead encapsulated in plant viral VLPs. mRNAs encapsulated this way have no cold chain requirements, unlike LNPs, and thus can be accessible to a great many people. They are also much smaller in diameter than LNPs, and thus are able to enter solid tumors more easily.

These advantages have been demonstrated using a TMV-based platform in which a self-amplifying Nodamura virus replicon encoding the SARS-CoV-2 receptor-binding domain (RBD) was encapsulated within TMV-derived plant virus nanoparticles (PVNPs) [201]. Following *in vitro* transcription, the replicon RNA was packaged with purified TMV coat protein to produce recombinant nanoparticles capable of delivering the RNA vaccine cargo. The encapsulated self-amplifying RNA drove efficient antigen expression and induced virus-neutralizing antibodies in animal models. Importantly, incorporation of the self-replicating replicon substantially enhanced antigen production, yielding approximately tenfold greater transcript accumulation than nonreplicating RNA controls.

The applicability of this approach extends beyond COVID-19 vaccines. TMV-based PVNPs have also been employed to deliver a self-amplifying RNA vaccine encoding a mutated HPV16 E7 oncoprotein, generating E7-specific antibody responses in animal studies [202]. In parallel, CPMV nanoparticles have been functionalized with surface-conjugated affimers that target specific cell populations, thereby enhancing the specificity and efficiency of mRNA delivery to selected tissues [203,204].

Collectively, these studies highlight the multifunctional nature of PVNPs as RNA delivery vehicles. By combining protection of RNA cargo with intrinsic adjuvant properties, scalable manufacturing, and opportunities for cell-specific targeting, PVNPs offer several advantages over conventional delivery systems. Their capacity to encapsulate self-amplifying RNA, enhance antigen expression, and incorporate targeting ligands positions them as promising next-generation platforms for mRNA vaccines and therapeutics against both infectious diseases and cancer.

Plant-derived vaccines have been generated targeting global diseases such as influenza and hepatitis. A majority of influenza and hepatitis viruses described in this review disproportionately impact developing nations. The advancement of new vaccines comes at a price that is challenging for resource-poor regions that are most in need of these vaccines. Additionally, plant-made vaccines are efficacious in precluding zoonotic diseases in addition to being low-cost and affordable vaccine options. Further, these vaccines are facile to scale up and can be stored at ambient temperatures. HBsAg has been expressed in maize plants wherein it has been reported to be stable at ambient room temperature for up to a year. Additionally, defatting the grain improved its stability at 45 °C temperature for up to 1 month [205]. Mett et al., 2008, reported the plant-based transient expression and analysis of GD and SD domains of HA of influenza H3N2 virus 16 produced as fusions with the lichenase LicKM, an engineered thermostable enzyme, and amino acids 38–469 of NA antigen from this virus [206,207,208]. Upon testing in ferrets, this vaccine candidate representing the HA and NA antigens was found to be highly immunogenic and thermostable (65 °C for over 10 min) and afforded protection against challenge with the H3N2 virus. Edible tissues of plants for vaccine expression afford a facile process wherein the harvested tissue is precisely formulated into oral capsules or powder [209]. The storage organelles of plants often inherently compartmentalize the respective antigens. For instance, transgenic rice seeds/grains have been shown to express cholera toxin B subunit in which this antigen was sequestered in protein bodies protecting it from the acidic environment in the stomach and enabled its deliverance to mucosal tissues in the gut. Owing to such stability, this matrix called MucoRice-CTB is formulated as a powder facilitating its storage and transportation at ambient room temperature, dispensing with the need for refrigeration [210]. Taken together, these characteristics may decrease the necessity for purification of antigens and cold chain storage.

Sustainable enduring cooperation and collaborations with global organizations, including governments, the WHO and World Bank, as well as others, need to be established to ensure the success of plant molecular farming and to become a productive mainstream system for vaccine development and manufacture. The studies presented in this manuscript have explored the current status of plant-made vaccines against influenza and hepatitis viruses, even to the point of commercialization. While some work is certainly required in this area of focus, it seems that the usage of plants as production factories for the large-scale combat of these global diseases is only a matter of time.

## Figures and Tables

**Table 1:** Genomic and structural characteristics of influenza and hepatitis viruses.

Virus Group	Virus	Family	Genome Type	Genomic Structure & Key Features
Influenza	Influenza A Virus (IAV) (Type A)	Orthomyxoviridae	ssRNA (−)	Segmented (8 segments), enveloped; highly diverse.
	Influenza B Virus (IBV) (Type B)	Orthomyxoviridae	ssRNA (−)	Segmented (7 segments), enveloped; less diverse than A.
	Influenza C Virus (ICV) (Type C)	Orthomyxoviridae	ssRNA (−)	Segmented (6 segments), non-enveloped/less complex.
	Influenza D Virus (IDV) (Type D)	Orthomyxoviridae	ssRNA (−)	Segmented; primarily infects swine/cattle.
Hepatitis	HAV	Picornaviridae	ssRNA (+)	Linear, non-enveloped; contains a 5′ VPg protein.
	HBV	Hepadnaviridae	dsDNA (partial)	Circular, partially double-stranded; replicates via reverse transcription.
	HCV	Flaviviridae	ssRNA (+)	Linear, enveloped; encodes a single polyprotein.
	HDV	Deltaviridae	ssRNA (−)	Small, covalently closed circular RNA; requires HBV for replication.
	HEV	Hepeviridae	ssRNA (+)	Linear, non-enveloped; contains three distinct ORFs.
	HGV	Flaviviridae	ssRNA (+)	Linear, enveloped; closely related to HCV architecture.

**Table 2:** Influenza and hepatitis virus antigens for production of VLPs.

Name of Virus	Epitope of Virus	Expression System	Ref
Influenza viruses	Hemagglutinin (HA), Neuraminidase	*Nicotiana benthamiana*	[44,48,49,50,51,52,53]
Hepatitis viruses	Small-HbsAg (HbsAgS), Core, E1, E2, and P7	Yeast cells, *E. coli*, Mammalian cells (CHO)	[45,54,55]

**Table 5:** PV-VLPs displaying foreign antigens of influenza viruses.

Plant Viral Vector	Target Virus	Displayed Antigen	Outcomes	Ref
Tobacco mosaic virus (TMV)	Influenza	M2e epitope, Hemagglutinin (HA)	Significant difference between IgG1 and IgG2a (IgG1/IgG2a = 3.2); robust IgG, IgM, and IgA responses	[169]
Physalis mottle virus (PhMV)	Influenza	M2e epitope	High levels of M2e-specific IgG antibodies in serum	[67]
Potato virus X	Influenza	M2e epitope	High levels of M2e-specific IgG antibodies in serum and mucosal secretions	[135]
Cowpea mosaic virus (CPMV)	Influenza	M2e epitope, Hemagglutinin (HA)	High levels of M2e-specific serum antibodies, the robust population of IL-2 + TNFα + IFNγ- CD4 + T cells	[170,171]
Maize mosaic virus (MaMV)	Influenza	M2e epitope	Cross-reactive immunoglobulin (total IgG and IgG2a) in mice and in dogs (tot IgG)	[172]

**Table 6:** PV-VLPs displaying foreign antigens for hepatitis viruses.

Plant Viral Vector	Target Virus	Displayed Antigen	Outcomes	Ref
Papaya mosaic virus (PapMV)	Hepatitis C virus (HCV)	E2 antigenic epitope	The antibody profile (production of IgG1, IgG2a, IgG2b, IgG3) suggests a Th1/Th2 response	[139]
Peanut yellow mosaic virus (PYV)	Hepatitis B virus (HBV)	PreS1 domain	Strong anti-preS1 immune response, even in the absence of adjuvants	[160]
Potato virus X (PVX)	Hepatitis C virus (HCV)	Envelope protein E2 (R9 peptide)	Anti-R9 IgG titers of up to 1:50,000. Monoclonal anti-R9 antibodies were obtained from the spleen of a mouse	[173]
Cucumber mosaic virus (CMV)	Hepatitis C virus (HCV)	HCV envelope protein E2 (R9 mimotope)	Inducing a significant release of interferon (IFN)-gamma, interleukin (IL)-12 p70 and IL-15 by lymphomonocyte cultures	[174]

## Data Availability

No new data were created or analyzed in this study. Data sharing is not applicable to this article.
